# P-5. Human Papillomavirus Vaccination Coverage Among Girls and Boys in the United States: A Birth Year Cohort Analysis of the National Immunization Survey-Teen, 2016–2022

**DOI:** 10.1093/ofid/ofae631.216

**Published:** 2025-01-29

**Authors:** Sarah Meyer, David Nmn Yankey, Laurie Elam-Evans, Sandy Pingali, Shannon K Stokley, James A Singleton, Ponesai Nyika

**Affiliations:** Centers for Disease Control and Prevention, Atlanta, Georgia; CDC/NCIRD/ISD/SEB, ATLANTA, Georgia; CDC, Atlanta, GA; Centers for Disease Control and Prevention, Atlanta, Georgia; Immunization Services Division, National Center for Immunization and Respiratory Disease, CDC, Atlanta, Georgia; Centers for Disease Control and Prevention, Atlanta, Georgia; Centers for Disease Control and Prevention (CDC), Atlanta, Georgia

## Abstract

**Background:**

Human papillomavirus (HPV) causes approximately 37,000 cancers in the United States annually. HPV vaccine, recommended by CDC at age 11 or 12 years, prevents more than 90% of HPV-attributable cancers. We aim to identify coverage gaps to guide targeted interventions.

**Figure:**
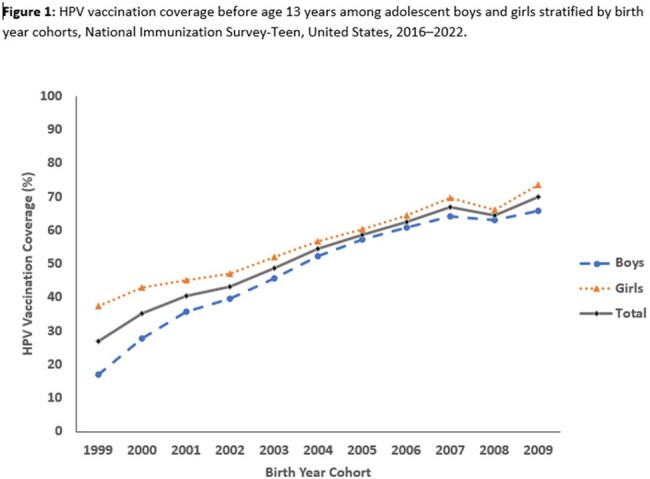

**Methods:**

We conducted a birth cohort analysis among adolescents born 1999-2009 using National Immunization Survey-Teen (NIS-Teen), a random-digit dialed telephone survey which also collects vaccination data from providers. We analyzed 131,553 records from 2016 to 2022 NIS-Teen data to determine: 1) trends in coverage with ≥ 1 HPV vaccine dose before age 13 years (on-time initiation), 2) cumulative coverage from 13 to 17 years (catch-up), 3) on-time HPV vaccination up to date status (all recommended doses before age 13 years), 4) missed vaccination opportunities (provider visits before age 13 years where HPV unvaccinated adolescents received other recommended vaccine but not HPV vaccine), and achievable coverage if HPV vaccination opportunities were not missed. Regression analysis provided the average percentage increase in coverage across birth cohorts. Kaplan-Meier method provided cumulative HPV vaccination coverage from age 13 to 17 years, stratified by birth cohorts.

**Figure:**
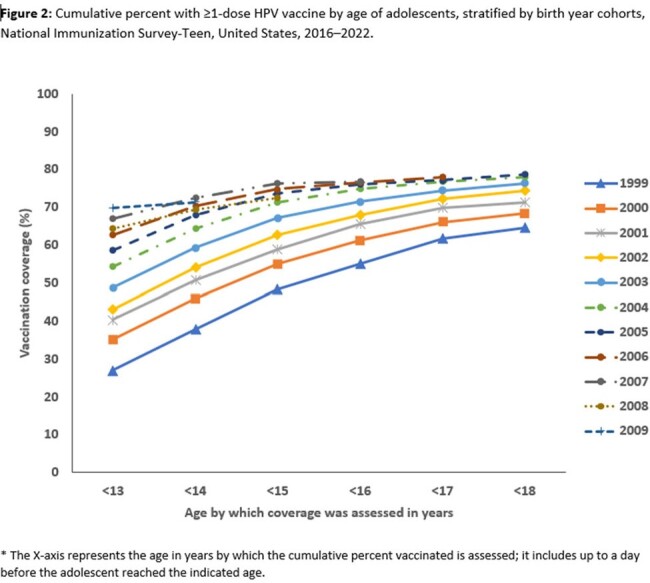

**Results:**

HPV vaccination coverage before 13 years increased from 27.0% among adolescents born in 1999 to 69.8% among those born in 2009. Coverage for girls increased from 37.4% to 73.4%; coverage for boys increased from 16.9% to 65.9%. Overall, coverage increased from 33.1% before age 13 years to 74.9% before age 18 years. HPV vaccination up to date status increase from 10.3% among adolescents in 1999 to 42.2% among those born in 2009. Among the 38,568 (29.3%) who had not received any HPV vaccination, 31,513 (82.5%) had ≥ 1 missed HPV vaccination opportunity. The potential achievable vaccination coverage if opportunities were not missed was 94.8%.

**Figure:**
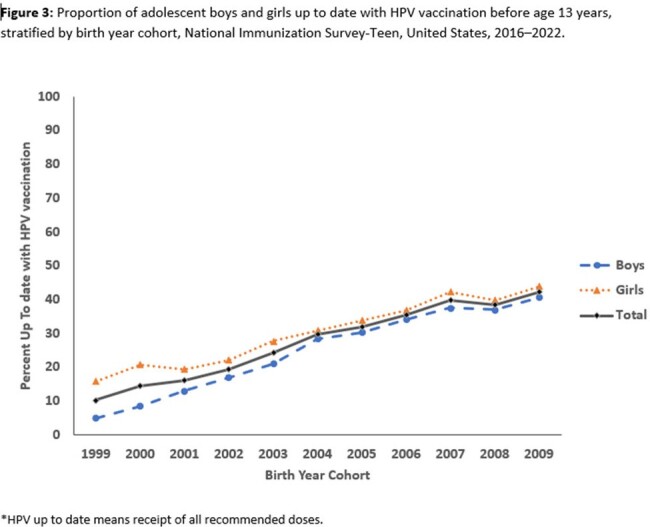

**Conclusion:**

Coverage with on-time HPV vaccination and HPV up to date increased by birth cohort among adolescents born 1999–2009 but remains suboptimal. Low uptake increases risk for HPV cancers. Opportunities for HPV vaccination before age 13 years are being missed and can be reduced by effective provider recommendations for HPV vaccination and by administering all recommended vaccines during the same visit.

**Disclosures:**

**All Authors**: No reported disclosures

